# EGF receptor stimulation shifts breast cancer cell glucose metabolism toward glycolytic flux through PI3 kinase signaling

**DOI:** 10.1371/journal.pone.0221294

**Published:** 2019-09-18

**Authors:** Kyung-Ho Jung, Eun Jeong Lee, Jin Won Park, Jin Hee Lee, Seung Hwan Moon, Young Seok Cho, Kyung-Han Lee

**Affiliations:** 1 Department of Nuclear Medicine, Samsung Medical Center, Sungkyunkwan University School of Medicine, Seoul, Korea; 2 Department of Health Science and Technology, SAIHST, Sungkyunkwan University, Seoul, Korea; 3 Department of Nuclear Medicine, Seoul Medical Center, Seoul, Korea; Universita degli Studi della Campania Luigi Vanvitelli, ITALY

## Abstract

Breast cancers that express epidermal growth factor (EGF) receptors (EGFRs) are associated with poor prognosis. Our group recently showed in breast cancer patients that EGFR expression is strongly correlated with high tumor uptake of the glucose analogue, ^18^F-fluorodeoxyglucose (FDG). Here, we explored the cellular mechanism and signaling pathways that can explain the relation between EGFR and breast cancer cell glucose metabolism. FDG uptake, lactate production and hexokinase (HK) activity were measured, and proliferation assays and western blots were performed. EGF stimulated an increase of FDG uptake in EGFR-positive T47D and MDA-MB-468 cells, but not in MCF-7 cells. In T47D cells, the effect was dose-dependent and was accompanied by increased lactate production, indicating a shift toward glycolytic flux. This metabolic response occurred through enhanced HK activity and upregulated glucose transporter 1 (GLUT1) expression. EGFR stimulation also increased T47D cell proliferation. Blocking EGFR activation with BIBX1382 or gefitinib completely abolished both FDG uptake and proliferation effects. EGFR stimulation induced MAP kinase (MAPK) and PI3 kinase (PI3K) activation. Increased cell proliferation by EGFR stimulation was completely abolished by MAPK inhibition with PD98059 or by PI3K inhibition with LY294002. Increased FDG uptake was also completely abrogated by PI3K inhibition but was uninfluenced by MAPK inhibition. These findings suggest that the association between breast tumor EGFR expression and high FDG uptake might be contributed by stimulation of the PI3K pathway downstream of EGFR activation. This was in contrast to EGFR-mediated cell proliferation that required MAPK as well as PI3K signaling.

## Introduction

Breast cancer is a major cause of cancer-related death in women [1]. In these tumors, the status of estrogen receptors (ER), progesterone receptors (PR) and human epidermal growth factor receptor 2 (HER2) are well-recognized predictors of treatment response and prognosis [2, 3]. Another receptor frequently overexpressed in triple-negative breast cancers (TNBCs), a subgroup associated with particularly poor treatment response and outcomes, is the epidermal growth factor receptor (EGFR) [4–6]. EGFR expression has been associated with poor patient prognosis [4, 5], and this is observed not only in TNBC but also in non-TNBC subtypes [4–7].

Positron emission tomography/computed tomography (PET/CT) imaging with ^18^F-fluorodeoxyglucose (FDG) is often used in patients with breast cancer [8, 9]. Breast cancer cells with high glucose metabolism are associated with more aggressive behavior and greater invasiveness [10, 11]. Accordingly, the magnitude of breast tumor FDG uptake offers useful information for decision making and prognostication [8, 9], as well as for predicting response to therapy [12, 13]. Our group recently discovered that breast tumor FDG uptake is more strongly influenced by EGFR status than by other major biomarkers [14]. In various cancers, FDG PET may also allow monitoring of tumor response to therapies targeting the EGFR pathway [15–17].

The EGFR is a member of the ErbB family of membrane tyrosine-kinase receptors. Activation of this receptor and its downstream pathways have been shown to mediate breast cancer cell migration and proliferation and protection from apoptosis [18]. Signaling from EGFR also represents a major mechanism through which breast tumors that are initially responsive to endocrine therapy acquire resistance [19, 20]. There is rising interest in understanding the metabolic plasticity of cancer cells and designing novel therapeutic options that target their metabolic characteristics [21]. Therefore, a better understanding of the mechanism and signaling pathways through which EGFR influences breast cancer cell glucose metabolism would benefit these endeavors.

In this study, we thus evaluated how EGFR activation influences breast cancer cell glucose metabolism and proliferation. We further investigated the underlying mechanisms for the responses and the roles of the mitogen-activated protein kinase (MAPK) and phosphoinositide 3-kinase (PI3K) pathways.

## Materials and methods

### Reagents

RPMI-1640, fetal bovine serum, and phosphate buffered saline (PBS) were obtained from Lonza (Basel, Switzerland). Phenol red-free RPMI-1640, antibiotics, and Trypsin-EDTA were from Gibco BRL. The specific EGFR inhibitor BIBX1382 was from Calbiochem (La Jolla, CA). Goat antihuman p-PI3K antibody (p85a, Tyr508) was from Santa Cruz Biotechnology (Santa Cruz, CA). Rabbit antihuman EGFR antibody, mouse antihuman p-EGFR antibody (Tyr1068), ERK2 antibody, p-MAPK antibody (p44/p42, Thr202/Tyr204), rabbit antihuman PI3K antibody and horseradish peroxidase (HRP)-conjugated secondary anti-rabbit, anti-mouse, and anti-goat IgG antibodies were from Cell Signaling Technology (Danvers, MA). Rabbit antihuman GLUT1 antibody was from Dako. All other reagents were obtained from Sigma Chemicals (St. Louis, MO). The specific PI3K inhibitors wortmannin and LY294002, the specific MAPK inhibitor PD98059, the selective EGFR kinase inhibitor BIBX 1382 and gefitinib, and the protein synthesis inhibitor cycloheximide were prepared as stocks in dimethyl sulfoxide (DMSO). Doses used were 100 nM for cycloheximide, 5 μM for BIBX1382, 5 μM for gefitinib, 200 nM for wortmannin, 10 μM for LY294002, and 50 μM for PD98059. Ethanol and DMSO vehicles were used as controls as indicated.

### Cell culture and stimulation

Human breast carcinoma T47D cells that are EGFR and ER positive, MDA-MB-468 cells that are EGFR positive but ER-negative, and MCF-7 cells that are EGFR weakly positive and ER positive were obtained from the American Type Culture Collection. Cells were maintained in RPMI-1640 supplemented with 10% fetal bovine serum and penicillin-streptomycin in a 5% CO_2_ incubator at 37°C. Cells were split 3 days before experiments and seeded with phenol red-free RPMI-1640 medium containing 5% charcoal-stripped serum (CSS). Experiments were performed when cell confluence reached approximately 80%. Cells were stimulated by addition of EGF to culture medium. Inhibitors were added to the culture medium 30 min prior to EGF stimulation.

### Cell proliferation measurement

Cell proliferation was assessed by sulforhodamine B (SRB) colorimetric assays as previously described [22]. Briefly, cells were suspended in phenol red-free RPMI-1640 with 5% CSS and seeded to 96-well microplates with a density of 3.0 × 10^3^ cells per well. Plates were incubated at 37°C in a humidified incubator with 5% CO_2_ until cell attachment was complete. Cells were fixed by adding 100 μL of cold 10% (wt/vol) trichloroacetic acid to each well without removing culture media. After incubation at 4°C for 1 h, plates were washed four times with slow-running tap water and dried completely. For staining, 50 μL of 0.04% (wt/vol) SRB solution was added, and plates were left at room temperature for 30 min and then quickly rinsed four times with 1% (vol/vol) acetic acid to remove unbound dye. After complete drying, 200 μL of 10 mM Tris base solution (pH 10.5) was added and plates were shaken on a gyratory shaker for 5 min to solubilize the protein-bound dye. Each sample was measured for OD at 510 nm on a microplate reader.

### Glucose uptake measurement

For glucose uptake measurements, cells were incubated at 37°C for 40 min with 185 kBq (5 μCi) FDG added to the culture medium. After rapid washing twice with cold PBS, cells were lysed with 0.1N NaOH and measured for radioactivity on a high-energy γ-counter (Wallac). Each sample was then measured for protein content by Bradford protein assays, and uptake results were expressed as protein content-corrected counts relative to those of the control cells.

### Lactate production measurement

Culture media and cell lysates were used to measure extruded and intracellular levels of lactate formations, respectively. Cells were seeded to 100 mm plates with phenol red–free RPMI-1640 containing 5% CSS. At appropriate time points, culture media were transferred to new tubes and cells were lysed in 300 μL of distilled water. Quantitative determination of L-lactate in the samples was performed using a Cobas assay kit (Roche/Hitachi, Mannheim, Germany) following the manufacturer’s instructions. The assay uses an enzymatic reaction that converts lactate to pyruvate and hydrogen peroxide. Hydrogen peroxide then undergoes an enzymatic reaction to generate a colored dye that is measured by a Roche/Hitachi analyzer. Final results were expressed as percent lactate concentration relative to that of the control cells.

### Total hexokinase activity measurement

Cell membranes were disrupted by syringe pumping twenty times on ice. Supernatants containing free and mitochondria-bound hexokinase were obtained by centrifugation at 1,000g at 4°C for 10 min. A buffer comprised of the homogenization buffer containing 0.5 mmol/L glucose, 5 mmol/L adenosine triphosphate, 0.25 mmol/L reduced nicotinamide adenine dinucleotide phosphate, and 6 units glucose-6-phosphate dehydrogenase was preincubated at 20°C for 15 min. Samples were then added, and the reaction mixture was measured for absorbance. Hexokinase activity was determined from a standard curve, with 1 unit defined as the enzyme activity that phosphorylates 1 μmol/L of glucose per min at 20°C. Results were expressed as protein-corrected enzyme activity relative to that of the control cells.

### Immunoblotting for p-EGFR, p-PI3K, p-MAPK, and membrane GLUT1

For immunoblotting of p-EGFR, p-PI3K, and p-MAPK, cells were washed with cold PBS and solubilized at 4°C for 15 min with shaking in 500 μL PRO-PREP Protein Extraction Solution (iNtRON Biotechnology, Inc.) and 1 mM sodium orthovanadate. After centrifugation at 14,000 rpm and 4°C for 10 min, the supernatant was measured for protein concentration. Protein (20 μg) boiled for 5 min was separated on a 10% polyacrylamide gel, membrane-transferred, and incubated with a polyclonal antibody against mouse p-EGFR (1:1,000 dilution), goat p-PI3K (p85a, Tyr508; 1:250 dilution), or rabbit p-MAPK (p44/p42, Thr202/Tyr204; 1:1,000 dilution). Immune-reactive protein was visualized by incubation with a secondary anti-mouse IgG antibody (1:2,000 dilution), anti-goat IgG antibody (1:2,000 dilution), or anti-rabbit IgG antibody (1:2,000 dilution), followed by 1 min soaking in ECL detection reagent and exposure to a high-performance chemiluminescence film. Immunoreactive protein band intensities were measured using a GS-800^TM^ calibrated densitometer and Quantity One^®^ software (Bio-Rad Laboratories, CA). After visualizing phosphorylated proteins, membranes underwent a stripping procedure and were re-incubated with antibodies against respective total proteins.

Plasma membrane GLUT1 protein was prepared as previously described [23]. Briefly, cells were treated with 10 strokes on a dounce homogenizer in HEPES buffer (20 mM 4-(2-hydroxyethyl)-1-piperazineethanesulfonic acid, pH 7.4, 1 mM ethylenediaminetetraacetic acid, and 250 mM sucrose) containing 5 mM benzamidine, 1 μM aprotinin, 1 μM leupeptin, 1 μM pepstatin, and 1 mM phenylmethylsulfonyl fluoride. After elimination of cell debris by 10 min of centrifugation at 14000 rpm and 4°C, supernatants were mixed with 1.5 mL lysis buffer containing 0.0856 g/mL sucrose, 10 mmol/L/mL HEPES, and 10 mmol/L/mL MgCl_2_, incubated at 4°C for 1 h, and centrifuged at 42,000 rpm for 60 min. The pellet was dissolved with a minimum volume of PBS, and 20 μg of membrane protein was separated on a 10% polyacrylamide gel, electroblotted to a hydrobond ECL nitrocellulose membrane (Amersham), and reacted with a polyclonal antibody against human GLUT1 (1:1,000 dilution). Immune reactive protein was visualized and measured as above using HRP–conjugated secondary anti-rabbit IgG antibody (1:5,000 dilution).

### Statistical analysis

All experiments were performed at least twice, and data are expressed as mean ± SD of duplicate or triplicate samples per group obtained from a single representative experiment. Comparisons were done by unpaired Student t-tests, and *p* values <0.05 were considered statistically significant.

## Results

### EGFR stimulation time-dependently augments T47D cell glucose uptake

**Fig 1:**
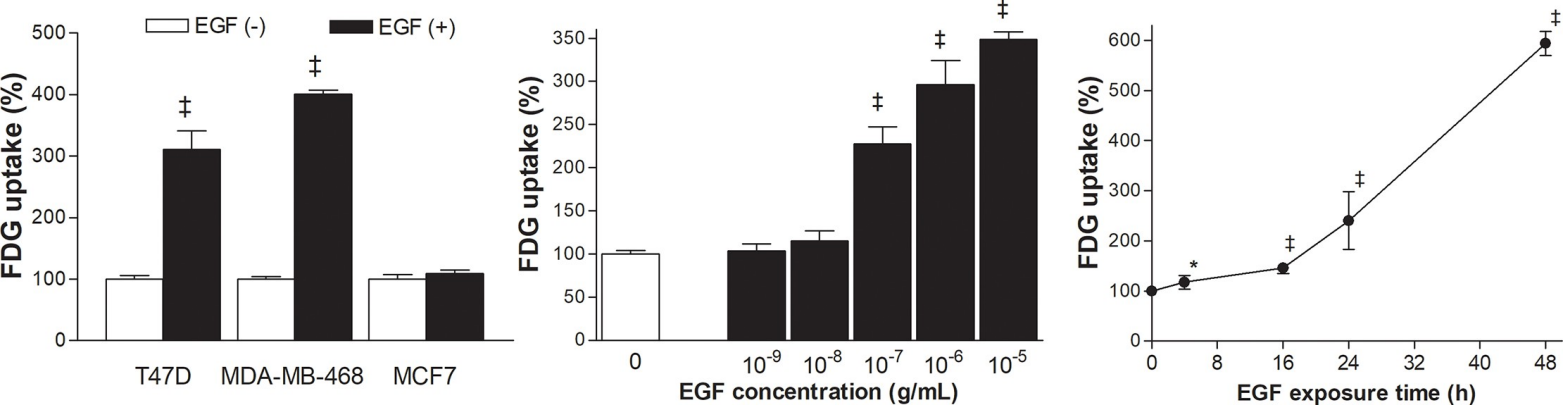
EGF dose- and time-dependently stimulates glucose uptake in T47D cells. (Left) Effects of 24 h treatment with 100 ng/mL EGF on FDG uptake of T47D, MDA-MB-468, and MCF-7 breast cancer cells. (Middle) Dose-dependent effects of 24 h EGF stimulation on T47D cell FDG uptake. (Right) Time course of FDG uptake in T47D cells stimulated with 100 ng/mL EGF. Results are mean ± SD % of control levels of triplicate (left and middle) or quadruplicate (right) samples obtained from a single experiment representative of two separate experiments. *, *P* <0.05; ‡, *P* <0.001, compared to untreated controls.

Exposure of T47D cells to 100 ng/mL EGF for 24 h stimulated a substantial augmentation of FDG uptake to 310.8 ± 30.1% of control level (*P* <0.001; Fig 1). The same treatment also increased FDG uptake in MDA-MB-468 cells that expressed high levels of EGFR (data not shown) to 400.6 ± 6.7% of controls (*P* <0.001; Fig 1). However, EGF did not influence FDG uptake of MCF-7 cells that expressed very little EGFR (Fig 1).

Time course experiments showed that stimulation of the FDG uptake effect by EGF began at 4 h (117.4 ± 13.6% of controls, *P* <0.05) and progressively increased to reach 594.3 ± 23.7% by 48 h (*P* <0.001; Fig 1). Dose-dependent experiments showed that EGF treatment, which began with a concentration of 0.1 μg/mL, increased FDG uptake within 24 h. Uptake was enhanced to 227.9 ± 19.6%, 296.1 ± 48.1%, and 348.5 ± 15.3% of control cells by 0.1, 1, and 10 μg/mL of EGF, respectively (all *P* <0.001; Fig 1).

### Shifting of metabolism toward glycolysis by upregulated hexokinase and GLUT1

**Fig 2:**
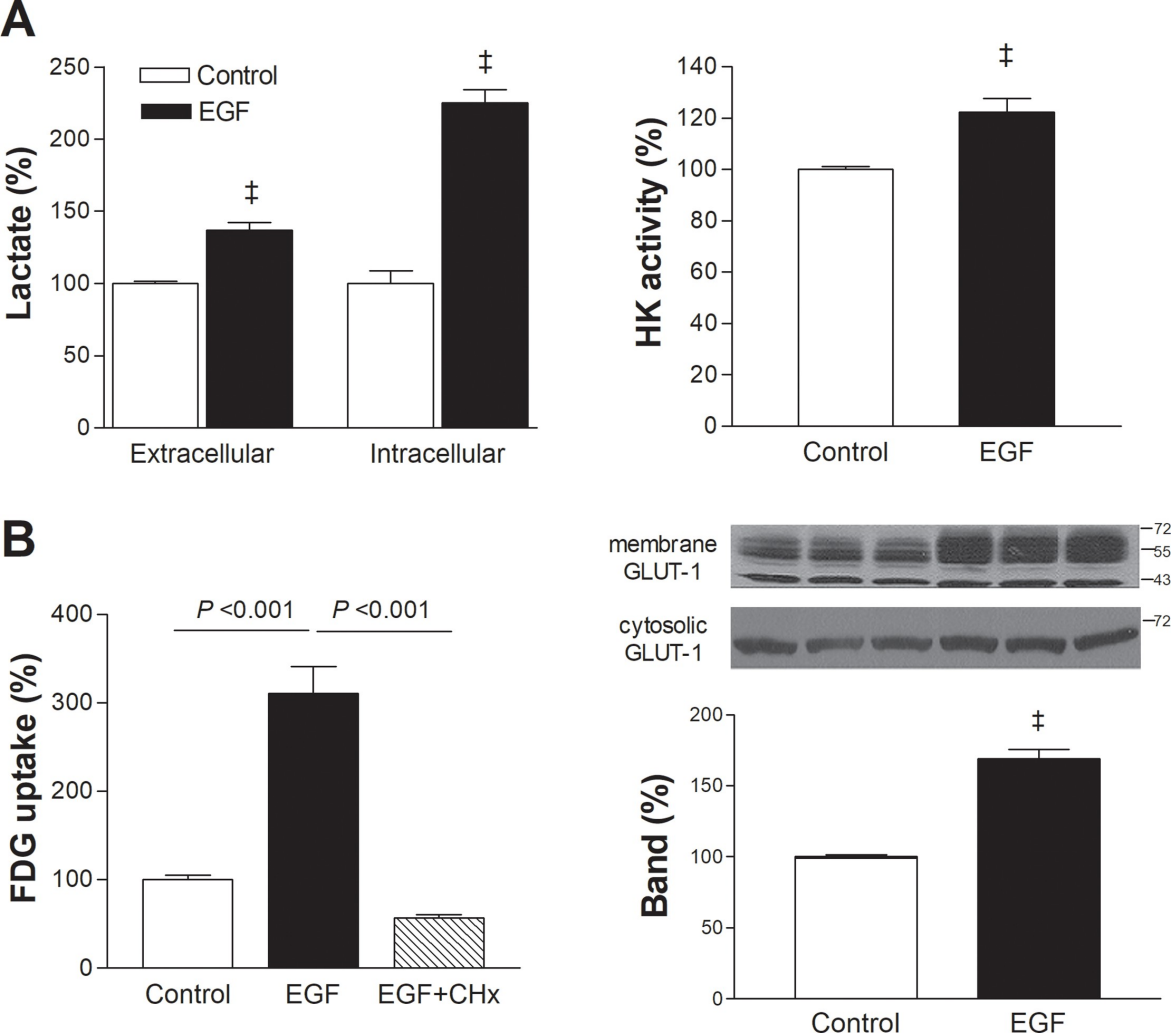
EGF augments T47D cell glycolytic metabolism through hexokinase and GLUT1 upregulation. (A) Effects of 24 h treatment with 100 ng/mL EGF on intracellular and extracellular lactate (left) and total cellular hexokinase (HK) activity (right). Results are mean ± SD of triplicate samples obtained from a single representative experiment (left) or mean ± SE of 6 samples per group obtained from 2 independent experiments (right). (B) Effect of cycloheximide (CHx; 100 nM) on EGF-stimulated FDG uptake (left) and membrane GLUT1 expression in EGF-treated cells (right). Results are mean ± SD % of control levels obtained from triplicate samples. ‡, *P* < 0.001, compared to controls.

T47D cells treated for 24 h with 100 ng/mL EGF showed increased intracellular and extracellular lactate concentrations that reached 225.2 ± 8.9% and 136.8 ± 5.3% of controls, respectively (both *P* <0.001; Fig 2A). This demonstrates that increased FDG uptake by EGFR stimulation results from a shift of glucose metabolism toward glycolytic flux.

EGF treatment caused an elevation of total cellular hexokinase activity to 122.4 ± 5.2% of control level (*P* <0.001; Fig 2A). The presence of 100 nM cycloheximide completely blocked the ability of EGF to stimulate FDG uptake (reduced to 57.2 ± 3.7% of controls), indicating a requirement of new protein biosynthesis for the metabolic effect (Fig 2B). Plasma membrane-expressed GLUT1 level was substantially increased to 169.1 ± 6.5% of controls by 24 h exposure to 100 ng/mL EGF while cytosolic GLUT1 expressions were not changed (*P* <0.001; Fig 2B right).

### EGF acutely and dose-dependently activates T47D cell EGFR

**Fig 3:**
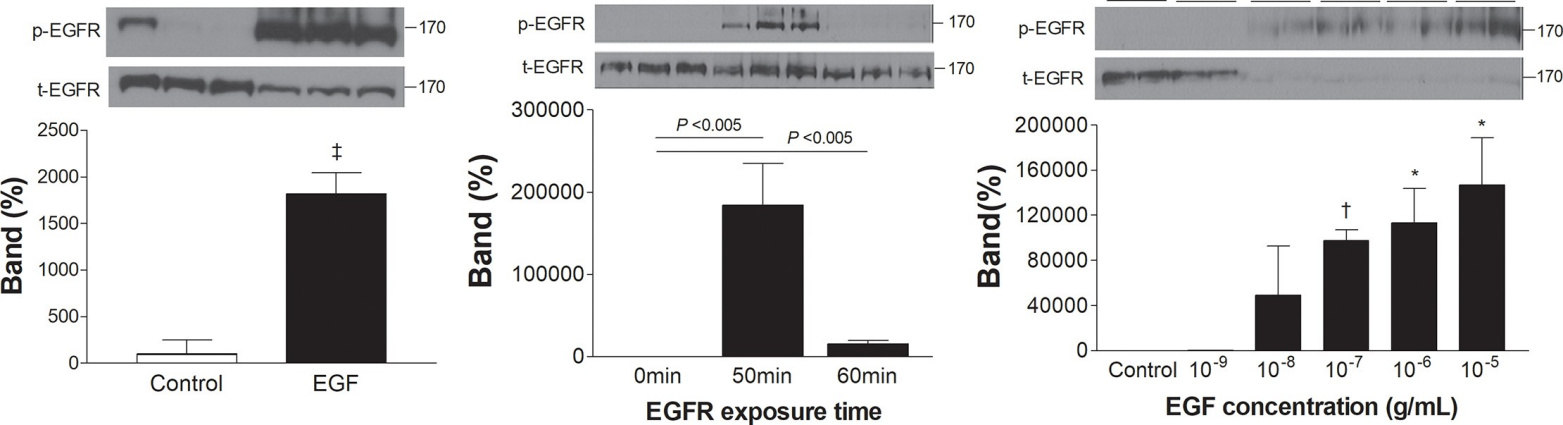
EGF acutely and dose-dependently activates T47D cell EGFR. Western blots and protein-band intensities of phosphorylated EGFR (p-EGFR) after EGF treatment. Cells were treated with 100 ng/mL EGF for 30 min (left) or 5 and 60 min (middle), or with graded EGF concentrations for 5 min (right). Bars are mean ± SD of % control obtained from triplicate (left and middle) or duplicate (right) samples. *, *P* <0.05; †, *P* <0.005, compared to controls.

Western blot analysis showed significant EGFR activation of T47D cells by 30 min treatment with 100 ng/mL EGF (Fig 3). Phospho-EGFR was substantially increased to 1839.8 ± 512.5-fold of controls by 5 min exposure to 100 ng/mL EGF and this effect was reversed after 60 min of exposure (Fig 3). Treatment with graded doses of EGF for 5 min revealed that EGFR activation began at a concentration of 10 ng/mL (Fig 3). This was lower than the EGF concentration of 100 ng/ml that began to stimulate FDG uptake. EGFR activation dose-dependently increased until an EGF concentration of 10 μg/mL (Fig 3).

### Effects of EGFR, MAPK and PI3K inhibition on EGF-stimulated glucose uptake

**Fig 4:**
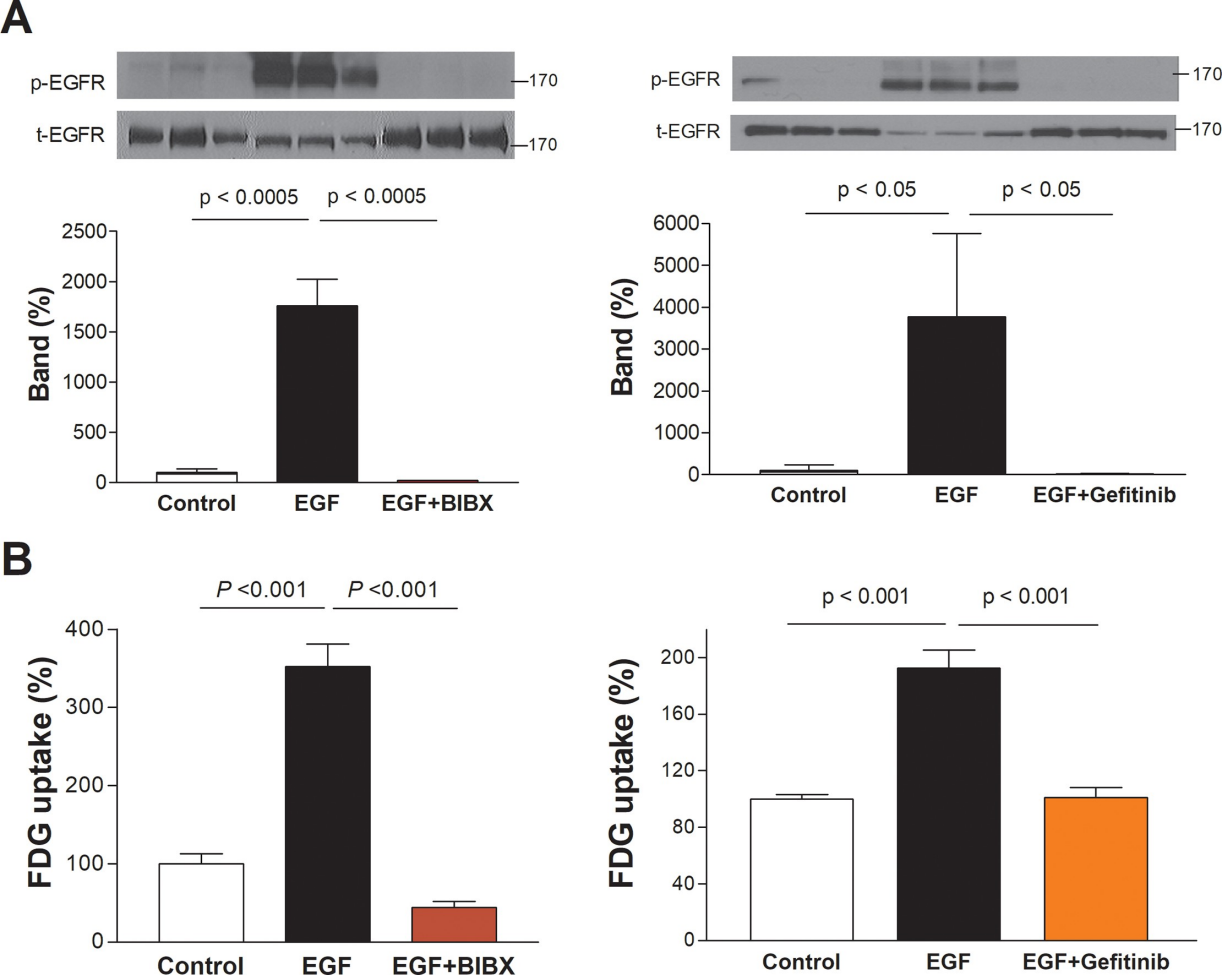
(A) Effects of BIBX1382 and gefitinib on EGFR activation (p-EGFR) and (B) FDG uptake. T47D cells were stimulated for 24 h with 100 ng/mL EGF in the absence or presence of 5 μM BIBX1382 (BIBX) and 5 μM gefitinib. Bars on the right are mean ± SD of % control obtained from triplicate samples.

EGFR activation in T47D cells was completely blocked by BIBX1382 as well as by gefitinib (Fig 4A). These EGFR inhibitors also completely abolished EGFR-stimulated FDG uptake to levels even lower than those of untreated controls (Fig 4B).

**Fig 5:**
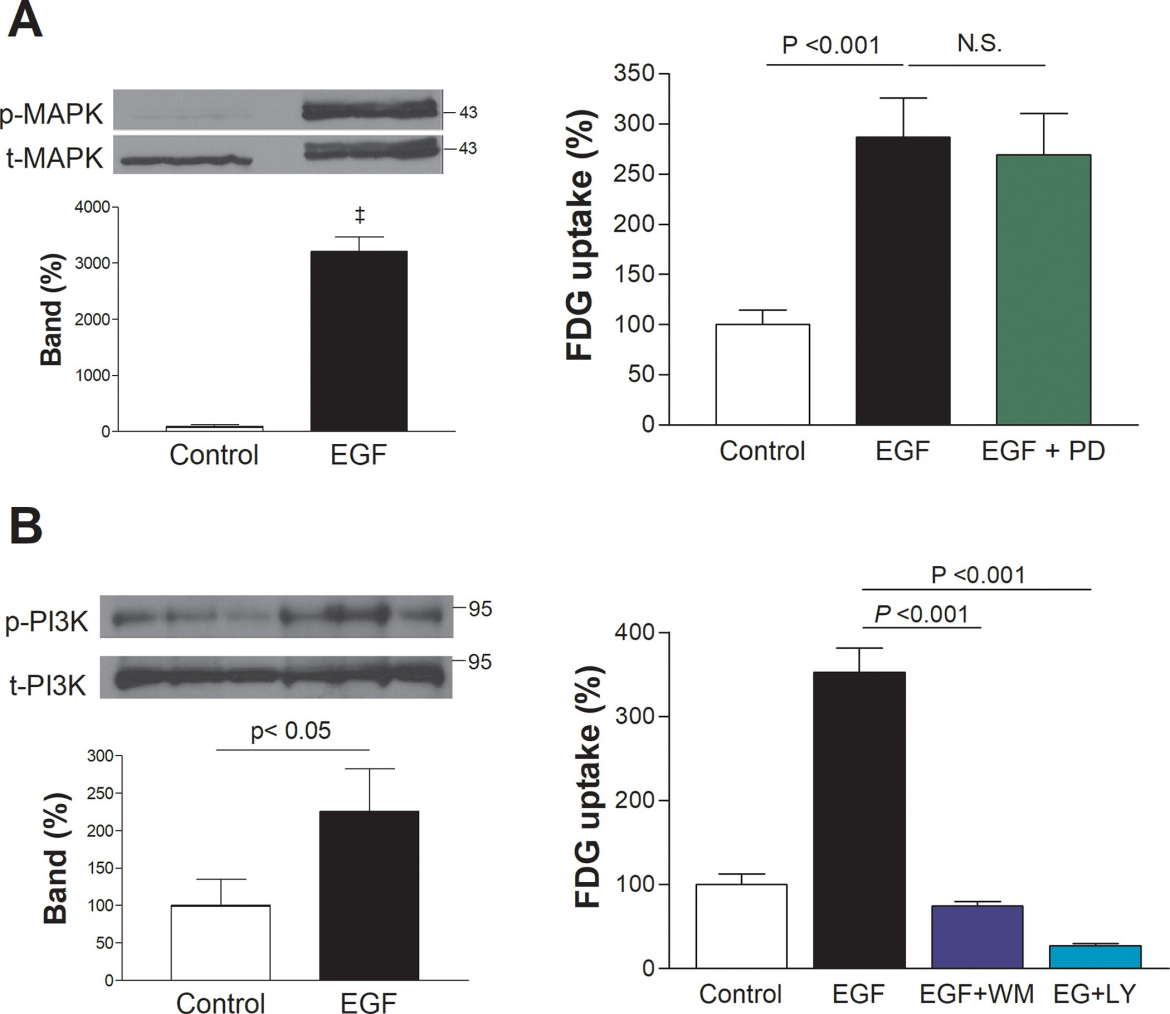
Effects of inhibition of MAPK and PI3K pathways in T47D cells. (A) Western blots and protein-band intensities of activated MAPK (p-MAPK) by 5 min treatment with 100 ng/mL EGF (left), and effect of 50 μM PD98059 (PD) on FDG uptake stimulated by 100 ng/mL EGF for 24 h (right). (B) PI3K activation (p-PI3K) by 5 min treatment with 100 ng/mL EGF (left), and effect of 200 nM wortmannin (WM) or 10 μM LY294002 (LY) on FDG uptake stimulated by 100 ng/mL EGF for 24 h (right). All bars are mean ± SD of % control obtained from triplicate samples. N.S., not significant.

Western blot analysis of T47D cells treated with 100 ng/mL EGF for 30 min demonstrated a clear increase of bands for phosphorylated (activated) MAPK (p-MAPK) at 42 and 44 kD (Fig 5A). However, PD98059 had no influence on EGFR-stimulated FDG uptake (Fig 5A), indicating that the metabolic effect is independent of the MAPK pathway.

T47D cells treated as above also demonstrated a clear increase in protein band for phosphorylated (activated) PI3K (p-PI3K) at approximately 85 kD (Fig 5B). In the presence of the specific PI3K inhibitors, wortmannin, or LY294002, FDG uptake in EGF-treated cells was markedly reduced to levels even lower than untreated controls (Fig 5B). This indicated the requirement of PI3K signaling for the metabolic effect of EGFR activation.

### Effects of MAPK and PI3K inhibition on EGFR-stimulated T47D cell proliferation

**Fig 6:**
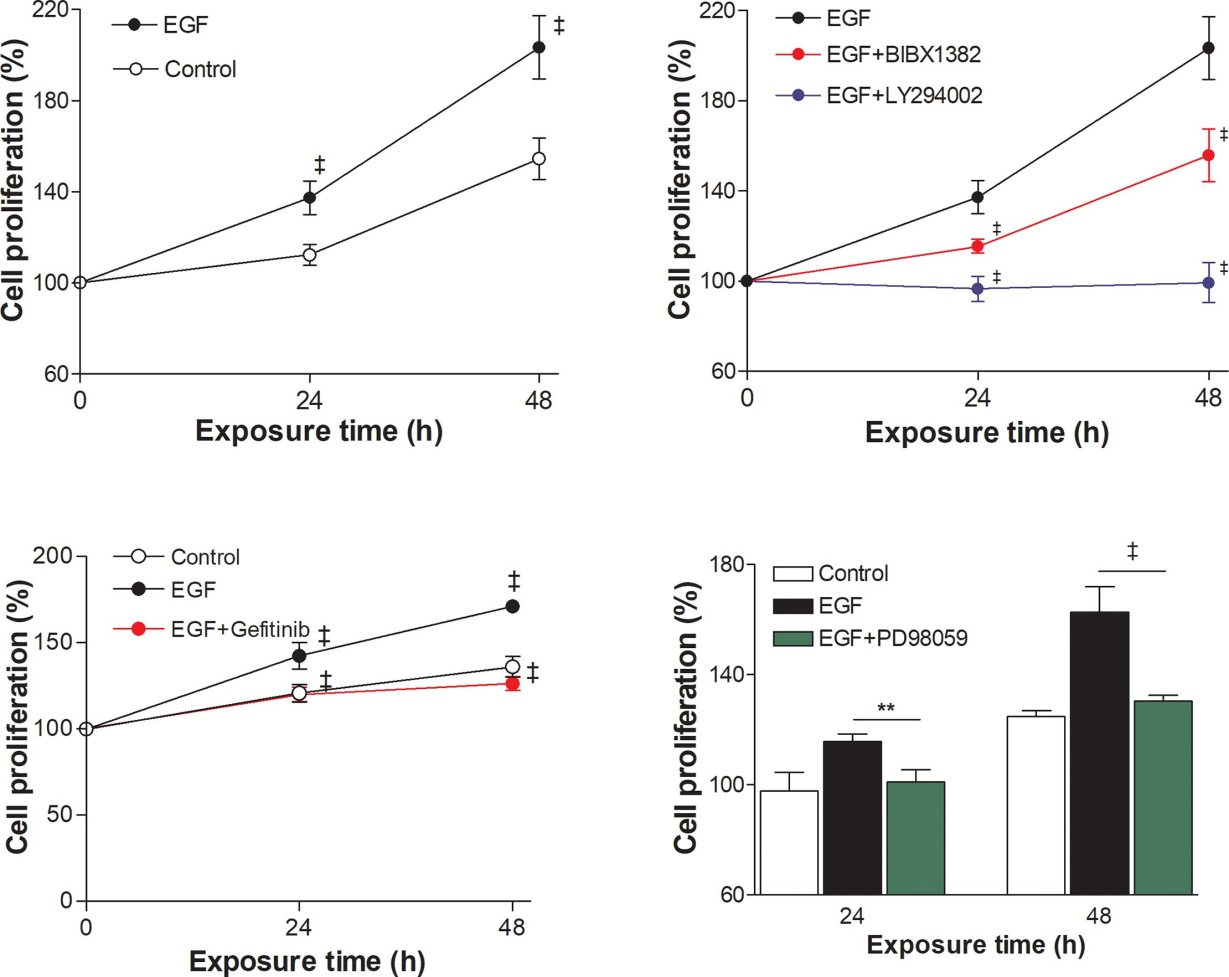
Effects of EGF and PI3K or MAPK inhibition on cell proliferation. Sulforhodamine B assays demonstrate increased T47D cell proliferation simulated by 100 ng/mL EGF (upper left). ‡, *P* <0.001, compared to controls at same time points. This effect was reduced by 5 μM BIBX1382 and was completely blocked by 10 μM LY294002 (upper right) and 5 μM gefitinib (lower left). PD98059 (50 μM) also completely suppressed the ability of EGF to stimulate T47D cell proliferation (right). **, *P* <0.01; ‡, *P* <0.001, compared to cells treated with EGF alone. Results are mean ± SD of % pretreatment levels from 6 (left and middle) or 3 (right) samples per group.

Proliferation assays showed that the cell content of untreated T47D cells increased to 112.2 ± 4.6% of baseline level by 24 h and 154.4 ± 9.1% by 48 h (Fig 6). Compared to these control levels, exposure to 100 ng/mL EGF significantly increased cell content to 137.3 ± 7.3% and 203.3 ± 13.9% of baseline level at 24 and 48 h, respectively (Fig 6).

The proliferative effect of EGF was suppressed to untreated control levels when EGFR activation was blocked by BIBX 1382 (Fig 6). EGFR kinase inhibition with gefitinib and PI3K inhibition with LY294002 even more completely blocked T47D cell proliferation (Fig 6). EGFR-stimulated proliferation was also completely blocked by MAPK inhibition with PD98059 (Fig 6). These results demonstrate that, dissimilar to its metabolic effect that requires only PI3K signaling, the proliferative effect of EGFR requires both PI3K and MAPK signaling.

## Discussion

Malignant cells are heavily dependent on glucose as their energy fuel, and the association between tumor growth and high glucose consumption is well established [24]. This study demonstrated that EGFR stimulation dose- and time-dependently augmented T47D breast cancer cell glucose uptake in a manner accompanied by increased lactate production, thus indicating enhanced glycolytic flux.

The metabolic effect of EGFR stimulation by its cognate ligand, EGF, occurred through upregulation of hexokinase activity and membrane GLUT-1 expression. Hexokinase constitutes the first step of the glycolytic pathway by catalyzing the conversion of glucose to glucose-6-phosphate. Augmented hexokinase activity by proliferative stimuli is thought to represent an adaptive response of rapidly replicating tumor cells to meet an increased energy demand [25]. We also found that membrane GLUT1 was significantly increased by EGF. GLUT1 is a facilitative hexose transporter widely overexpressed in breast cancers and mainly responsible for the high FDG uptake of T47D cells [26]. This response is similar to a previous observation that EGF transactivation led to increased GLUT1 expression in glomerular mesangial cells [26]. Our finding that protein synthesis inhibition with cycloheximide completely abolished the ability of EGF to stimulate FDG uptake points to the requirement for new protein biosynthesis. Previous studies reported apparent half-lives of 19 h for GLUT1 [27] and 20–22 h for hexokinase [28]. Thus, increased expression of GLUT1 and possibly also of hexokinase appears to contribute to EGF stimulation-induced glucose uptake.

The ErbB family ligands include EGF, transforming growth factor alpha (TGFα), amphiregulin, neuregulins, epiregulin, betacellulin, and heparin-binding EGF (HB-EGF). Based on their pattern of receptor recognition, EGF, amphiregulin and TGF-α are known to bind only the EGFR; HB-EGF, betacellulin and epiregulin bind EGFR and HER4; and the neuregulins bind HER3 and/or HER4 [29, 30]. Therefore, although EGFR can be activated by amphiregulin or TGF-α, EGF only acts on EGFR and not on other ErbBs. Similar to amphiregulin and TGF-α, EGF is reported to promote breast tumorigenesis through EGFR activation [31–33], and inhibition of EGF/EGFR signaling has been shown to exert anti-tumor effects for breast cancer [34, 35]. The EGF concentration of 100 ng/ml we used is within the range between 30 and 300 ng/ml that was observed in breast cyst fluids of adult women [36]. Furthermore, several other studies also used 100 ng/ml of EGF to investigate EGFR activation [31, 34, 35]. It should be noted, however, that different EGFR ligands possess different affinity-independent efficacy at the EGFR. More specifically, there are data indicating that amphiregulin and TGFα possess greater efficacy at the EGFR than does EGF, indicating that these ligands may have a more important role in tumorigenesis.

The increase of FDG uptake by EGF occurred in T47D cells and MDA-MB-468 cells that showed strong EGFR expression, but not in MCF-7 cells that showed low expression. A similar stimulatory effect of EGF on glycolytic flux in MDA-MB-468 cells but not MCF-7 cells was previously observed [37]. In T47D cells, EGF induced a substantial increase in activated EGFR.

The EGFR inhibitor BIBX 1382 and gefitinib completely abolished both EGFR activation and stimulation of glucose uptake. Thus, the metabolic effect of EGF treatment is mediated by EGFR activation. BIBX1382 is a cell-permeable pyrimidopyrimidine compound that acts as a potent and highly selective EGFR tyrosine kinase inhibitor [38]. BIBX1382 at 5 μM has been shown to markedly inhibit EGF-mediated activation of EGFR and down-stream AKT and ERK1/2 [39, 40]. BIBX1382 exhibits 1,000-fold greater selectivity for EGFR over ErbB-2 and shows little activity towards other potential targets including receptors for insulin-like growth factor-1, hepatocyte growth factor, and vascular endothelial growth factor-2, even at concentrations as high as 10 μM. Gefitinib is a highly specific EGFR tyrosine kinase inhibitor that interrupts EGFR signaling and exerts antitumor effects. In lung cancer cells, gefitinib at 5 μM was shown to completely suppress activation of EGFR, Akt and ERK1/2 [41, 42]. Even so, we cannot completely exclude the possibility that BIBX1382 and gefitinib at 5 μM may have suppressed targets other than EGFR in our experiments.

Importantly, EGFR stimulation also augmented the proliferation in T47D cells. Binding of EGF to EGFR can activate multiple intracellular signaling kinases including c-Src, MAPK and PI3K. Activation of these pathways can in turn result in phosphorylation of various cytoplasmic enzymes and transcription factors that regulate cell proliferation and survival [43]. EGFR functions through the formation of homodimers or heterodimers with other members of the ErbB family [38]. Therefore, the effects of EGF on breast cancer cells that we observed could very well have involved other ErbBs through heterodimer formation. Although EGFR does not contain a site that can directly recruit PI3K, this may be accomplished indirectly through ErbB3 or ErbB4 that contain PI3K binding sites [44]. Alternatively, activated EGFR might indirectly recruit PI3K via other signaling molecules such as growth factor receptor-bound protein 2, Src homology 2 domain containing transforming protein (shc) or signal transducer and activator of transcription 5 (STAT5) [45]. Furthermore, our findings that EGF began to stimulate FDG uptake at a concentration higher than that required to activate EGFR suggests the possibility that EGF-dependent phosphorylation of something other than EGFR, such as ErbB3 or ErbB4, could have stimulated PI3K signaling and glycolytic flux.

Our results confirmed significantly increased PI3K and MAPK activation by EGF. We therefore tested the effects of blocking PI3K and MAPK pathways on the proliferative and metabolic effects of EGFR stimulation. As a result, its effect on cell proliferation was completely abrogated by either MAPK or PI3K inhibition. Unlike proliferation, however, its ability to increase glucose uptake was completely abolished by PI3K inhibition but was totally uninfluenced by MAPK inhibition. Thus, EGFR-induced T47D cell proliferation was dependent on both PI3K and MAPK pathways, whereas its ability to shift metabolism toward glycolytic flux required PI3K but not MAPK activity. This might reflect that PI3K-mediated upregulation of glycolysis is necessary for MAPK-mediated cellular proliferation that is driven by EGFR signaling. Our findings that BIBX1382, gefitinib and LY294002 reduced EGFR-mediated FDG uptake might also have relevance on the therapeutic effects of newer drugs for breast cancer that target EGFR [37, 46] or the PI3K pathway [47].

Given the relatively large number of reports on the effect of EGF on breast tumor cell proliferation, this study did not attempt to probe deeper into the detailed signaling pathways that mediate this response. Rather, we focused on how EGFR activation (using EGF as stimuli) influences breast cancer cell glucose metabolism. This question was brought up by our recent observation that breast tumor uptake of FDG in human patients was strongly influenced by EGFR status. Proliferation was evaluated to see to what extent major signaling pathways was shared. Yet, failure to demonstrate experimental evidence of novel mechanisms for EGF-triggered breast tumor proliferation might be considered a limitation of the present study.

In conclusion, EGFR activation shifts T47D breast cancer cell glucose metabolism toward glycolytic flux by upregulating hexokinase activity and GLUT-1 expression. It also enhanced cell proliferation in a manner that required both PI3K and MAPK pathways, whereas stimulation of glycolytic metabolism was only dependent on the PI3K pathway.

## Supporting information

S1 File (XLSX)
